# Metabolomics in Sleep, Insomnia and Sleep Apnea

**DOI:** 10.3390/ijms21197244

**Published:** 2020-09-30

**Authors:** Elke Humer, Christoph Pieh, Georg Brandmayr

**Affiliations:** 1 Department for Psychotherapy and Biopsychosocial Health, Danube University Krems, 3500 Krems, Austria; christoph.pieh@donau-uni.ac.at; 2 Section for Artificial Intelligence and Decision Support, Medical University of Vienna, 1090 Vienna, Austria; georg.brandmayr@gmail.com

**Keywords:** metabolomics, sleep, mental disorders, insomnia, sleep apnea

## Abstract

Sleep-wake disorders are highly prevalent disorders, which can lead to negative effects on cognitive, emotional and interpersonal functioning, and can cause maladaptive metabolic changes. Recent studies support the notion that metabolic processes correlate with sleep. The study of metabolite biomarkers (metabolomics) in a large-scale manner offers unique opportunities to provide insights into the pathology of diseases by revealing alterations in metabolic pathways. This review aims to summarize the status of metabolomic analyses-based knowledge on sleep disorders and to present knowledge in understanding the metabolic role of sleep in psychiatric disorders. Overall, findings suggest that sleep-wake disorders lead to pronounced alterations in specific metabolic pathways, which might contribute to the association of sleep disorders with other psychiatric disorders and medical conditions. These alterations are mainly related to changes in the metabolism of branched-chain amino acids, as well as glucose and lipid metabolism. In insomnia, alterations in branched-chain amino acid and glucose metabolism were shown among studies. In obstructive sleep apnea, biomarkers related to lipid metabolism seem to be of special importance. Future studies are needed to examine severity, subtypes and treatment of sleep-wake disorders in the context of metabolite levels.

## 1. Introduction

Sleep is a pivotal operating state of the central nervous system, which controls organismal well-being and might be the most well-preserved activity throughout the evolutionary timescale [1,2]. Sleep occupies up to a third of the human lifespan [3] and represents one of the most important psychophysiological processes for brain function and mental health [2]. It is well-documented that sleep disorders are highly prevalent in patients with mental disorders and they can lead to adverse effects on cognitive, emotional and interpersonal functioning [4,5]. Furthermore, sleep is required to balance catabolic activities experienced during the wake time by switching to anabolic functions [6], which indicates the essential role of sleep in restorative functions [7]. A lack of sleep contributes to maladaptive changes causing metabolic disorders, such as hypertension, cardiovascular diseases, diabetes and obesity [8,9,10,11]. Insufficient sleep has been demonstrated to cause enhanced expression of genes related to oxidative stress and immune response [12]. Additionally, partial sleep deprivation has been related to activated DNA damage response and promoted cellular senescence [13]. Sleep plays a pivotal role in memory consolidation, with slow wave sleep (SWS) being assumed to significantly impact the consolidation of acquired memories, while rapid eye movement (REM) is thought to be important for the stabilization of formed memories [14]. Animal models and human studies revealed that the process of aging is accompanied by alterations in sleep-wake activities [15], with aging individuals being unable to sustain a sleep state and having an overall lower sleep efficiency [16,17]. Lower sleep efficiency likely contributes significantly to the memory impairments associated with aging. A lack of SWS has been shown to impair long-term memory in cognitively normal older adults [18]. Furthermore, the incidental development of Alzheimer’s disease has been linked to lack of sleep [19]. To sum up, healthy sleep is required for restoring function and vitality, maintaining immune function and promoting memory consolidation and stabilization. Problems with sleep can enhance the risk of psychiatric disorders, somatic disorders (i.e., heart disease, diabetes, metabolic syndrome), traffic accidents and memory problems, as well as impaired functioning in general [20,21].

Overall, many individuals are affected by sleep disorders. Insomnia has a prevalence of 10% to 15% in the general population. Sleep apnea represents the second most prevalent sleep disorder with about 10%, followed by other sleep disorders, such as restless legs syndrome and circadian rhythm disorders [22]. Growing evidence suggests that sleep disorders coexist with other psychiatric disorders and medical conditions, which are often interactive and bidirectional [23]. Overall, the latest version of the diagnostic and statistical manual of mental disorders (DSM-5) [24] recognizes the need for independent clinical attention to sleep-wake disorders and considers pathological and etiological factors [23]. Sleep-wake disorders encompass ten disorders or diagnostic groups [25]: insomnia disorder, hypersomnolence disorder, narcolepsy, breathing-related sleep disorders (i.e., obstructive sleep apnea hypopnea, central sleep apnea, sleep-related hypoventilation), circadian rhythm sleep-wake disorders, non–rapid eye movement (NREM) sleep arousal disorders, nightmare disorder, REM sleep behavior disorder, restless legs syndrome and substance-/medication-induced sleep disorder. Patients with these diagnoses typically express dissatisfaction regarding the quality, amount and timing of sleep. Daytime distress and impairment represent core symptoms typically resulting from all of these sleep-wake disorders [24].

Recent studies support the notion that metabolic processes correlate with sleep [26]. The global scale study of metabolite biomarkers (metabolomics) in a large metabolite bandwidth manner offers unique opportunities to capture phenotypic changes of sleep-wake disorders and provide biomarkers for their prevention, diagnosis and prognosis and treatment monitoring. In this review, we aim to address the present status of knowledge on sleep from metabolomics analysis and to present knowledge in understanding the metabolic role of sleep in psychiatric disorders. As not every diagnostic group has been investigated using metabolomics approaches, the following review summarizes metabolomics studies for insomnia and obstructive sleep apnea. In addition, the effect of sleep on the metabolome in healthy individuals exposed to sleep deprivation, sleep fragmentation or sleep restriction is reviewed.

The following chapters summarize studies on the use of metabolomics approaches to reveal metabolic changes occurring due to alterations in sleep-wake activity. Therefore, a literature search was conducted using the Scopus, PubMed, and APA Psycinfo databases. Research articles in scientific journals on experiments using animal models or human subjects were considered. The search was conducted in July 2020, with no limitations on the publication date. Articles were identified by searching for titles using the following search terms: “metabolom*” AND “sleep* OR insomnia* OR apnea”. Despite the strategic literature search strategy, the current study does not follow PRISMA (preferred reporting items for systematic reviews and meta-analyses) guidelines for systematic reviews (e.g., quality assessment of studies).

## 2. Insomnia Disorder

Insomnia is regarded as a disorder in itself that needs independent clinical attention [27]. Therefore, the DSM-5 does not differ between primary and comorbid insomnia [24]. The diagnostic criteria are: repeated difficulties (at least three times a week for at least three months) with sleep initiation, maintenance, consolidation or quality that occur despite ample opportunity to sleep and that result in significant distress or impairment in functioning [25].

Studies suggest that poor or decreased sleep causes metabolic changes in peripheral metabolism (Table 1). In individuals with a clinical diagnosis of insomnia, analysis of serum samples every 2 h over 48 h under diurnal lighting demonstrated that the general insomniac profile clearly differed from age-, sex-, and race-matched controls. More specifically, alterations in branched-chain amino acid (BCAA) metabolism and lactate peak timing and amplitudes were observed [28].

Overall, most studies conducted thus far imposed short-term sleep deprivation and/or sleep restriction protocols in normal subjects; however, these are not reflective of long-term sleep habits (i.e., chronic sleep loss and/or sleep timing and/or pathological sleep conditions such as insomnia). A recent study aimed to fill this gap by investigating real world sleep habits in insomnia in humans. More specifically, metabolomics was applied on fasting plasma samples from 277 individuals over the course of a year where participants kept to their normal sleep habits. Here, 64 metabolites were found to be associated with sleep timing, with representation from all classes detected [29]. Elevated levels of amino acid metabolites, such as BCAAs and gamma glutamyl dipeptides, were associated with later sleep timing and no significant sex differences were observed [29]. In this study, the authors were also able to compare profiles from insomniacs versus controls and observed elevated energy metabolites and reduced BCAA catabolic products in insomniacs, particularly during the night. Altered phases of rhythmic metabolites were also observed in the insomnia samples compared to the controls [28], further implying a role for sleep in maintaining temporal separation. These results are consistent with a hyperarousal hypothesis in insomnia, which has been observed at the cognitive level, with limited previous evidence in metabolism. Specifically, phase advances and increased catabolism of BCAAs along with altered glucose metabolism were observed during the night in insomniacs, both of which point to increased susceptibility to diabetes [28]. These studies taken together suggest a conserved role of BCAAs in altered glucose metabolism and in disrupted sleep.

Despite a lack of (biological) chronic insomnia models that might enable the identification of insomnia biomarkers, some biomarkers can be suggested. Among those, alterations in BCAA and glucose metabolism show similarities among studies [30,31,32].

Taken together, these changes suggest that bedtime metabolic activity is shifted towards catabolism in patients with insomnia [28].

## 3. Obstructive Sleep Apnea

Within the category of breathing-related sleep disorders, most research has been conducted on obstructive sleep apnea (OSA), which refers to a disturbance in breathing during sleep. The diagnosis is based on the number of episodes of upper airway collapse while sleeping, measured via polysomnography through overnight monitoring. Nocturnal breathing disturbances can cause snoring, gasping and breathing pauses during sleep, but also daytime impairments, such as sleepiness, fatigue or unrefreshing sleep despite sufficient opportunities to sleep [25]. Sleep apnea is strongly related to lifestyle diseases, with obesity representing a major risk factor for OSA; thus, the global increase in obesity likely contributes to the observed increase in OSA over time [34]. There is an association between OSA and mental health, showing comorbidities with depression, suicidal ideation, anxiety and post-traumatic stress disorders, as well as psychosis and schizophrenia [35,36]. Sleep apnea has also been found to be correlated with cardiac events, whereby the underlying metabolic mechanisms have barely been investigated so far [37].

Metabolomic approaches have been applied in the study of OSA, whereby they can be categorized into two types of studies, including (1) metabolomic studies of exhaled breath [38,39,40], and (2) studies of the blood and/or urine metabolome [41,42,43,44].

Exhaled breath is well suited for metabolomics analyses as its condensate can be easily obtained by cooling exhaled air from spontaneous tidal breathing and thus represents a non-invasive sampling method [45]. Although exhaled breath is comprised of 99.9% water, volatile organic compounds, non-volatile organic compounds and inorganic compounds are also included. Organic compounds comprise of amino acids and their derivatives, peptides, proteins, urea, organic acids, surfactants and macromolecules [46]. Studies show enhanced levels of inflammatory markers related to oxidative stress in the exhaled breath of OSA patients. Markers of chronic inflammation were enhanced, showing an increase in pro-inflammatory cytokines, while anti-inflammatory cytokines decreased [47]. Furthermore, elevations of toxic volatile organic compounds in patients with OSA were observed [38]. More specifically, aromatic hydrocarbons (phenylacetic acid, ethylbenzene, toluene and p-xylene), saturated hydrocarbons (hexane, octane, heptane, nonane and decane), acetone and isoprene were elevated, which have been suggested as potential biomarkers. However, studies have shown that changes in the metabolomics profile of exhaled breath might not be specific enough to differentiate between patients with different respiratory disorders, such as chronic obstructive pulmonary disease and OSA [42]. Therefore, the second group of studies (studies of the blood and/or urine metabolome) might provide a more specific characterization of OSA. 

The general notion is that metabolic biomarkers obtained from blood and/or urine samples are potentially useful in understanding OSA mechanisms and OSA management. A systematic review of individual metabolites detected by chromatography and/or mass spectrometry supports this conclusion [48]. Several metabolites related to lipid metabolism, amino acids, oxidative stress pathways, adrenergic/dopaminergic biomarkers and micromolecules have been reported to be altered in patients with OSA. Overall, studies conducted on blood or urine samples show evidence of perturbation of lipid metabolism [49], with an elevation of both fasting [50] and post-prandial lipid levels [51]. As post-prandial hypertriglyceridemia has been linked to elevated cardiovascular morbidity and mortality, an altered lipidomic equilibrium is suggested to be crucial for OSA-related cardiovascular disorders [52]. The accelerated atherogenesis in OSA is likely due to lipid metabolic dysregulation and dyslipidemia, as revealed in animal studies [53,54]. As reviewed by Xu et al. [55], at least 10 different metabolomics studies in clinical populations revealed plasma biomarkers of OSA. Overall, metabolites related to lipid-metabolism represented a significant fraction of reported biomarkers, comprising mainly lipids or related biomarkers. Those biomarkers included phospholipids (phosphatidylcholines, posphatidylserines, lysophosphatidylcholines, lysophosphatidic acid, phosphatidylethanolamine, sphingomyelins) [41,56] and circulating endocannabinoids [57]. Furthermore, differences in urine metabolome were observed, including changes in acylcarnitines, glycerophospholipids and sphingomyelins [58]. Further metabolites that have been suggested as OSA-biomarkers are acylcarnitine C14:1, sphingomyelin 18:1 and symmetric dimethylarginine [1]. Urine metabolomics analysis was also applied to differentiate between OSA, simple snorers and healthy controls [43]. A combination of six metabolites (4-hydroxypentenoic acid, arabinose, glycochenodeoxycholate-3-sulfate, isoleucine, serine and xanthine) was capable of distinguishing between individuals with OSA and without OSA. In addition, a combination of five metabolites (4-hydroxypentenoic acid, 5-dihydrotestosterone sulfate, serine, spermine and xanthine) enabled the differentiation of OSA-individuals from simple snorers. Elevated levels of BCAAs and altered insulin sensitivity have also been observed in children diagnosed with OSA [59]. These results also point to metabolic dysfunction and altered glucose metabolism in OSA patients, in addition to an increased risk of developing diabetes. These studies, taken together, suggest a conserved role of BCAAs in altered glucose metabolism and in disrupted sleep.

Although continuous positive airwave pressure (CPAP) is the treatment of choice for patients with OSA [60], there is a lack of research on its effect on metabolomic profiles. The application of traditional chemometric approaches suggests an improved glucose metabolism as indicated by an improved glycemic control and insulin resistance when compared to a control group [61]. The CPAP treatment has also been shown to improve lipid metabolism in pre-post comparisons, showing a decrease in total cholesterol and low-density lipoproteins, and an increase in high-density lipoproteins in patients subjected to CPAP treatment [62]. However, a study conducted in women observed no change of metabolites related to glucose and lipid metabolism (i.e., glucose, cholesterol, high-density lipoprotein, low-density lipoprotein, triglycerides, glycated hemoglobin and homeostasis model of insulin resistance) in OSA-patients receiving CPAP therapy as compared to a conservative treatment [60]. Therefore, metabolomics studies are needed to confirm findings suggesting beneficial effects of CPAP treatment on metabolism.

## 4. Sleep Deprivation

Sleep deprivation (no sleep opportunity), sleep restriction (partial sleep opportunity) and circadian clock disruption are associated with negative effects on mental health as well as metabolic disorders [63,64]. Table 2 depicts studies using metabolomics approaches in the study of sleep deprivation.

Differences in the plasma [30] and urine [65] metabolome have been found in sleep-deprived humans. In plasma, 27 of 171 metabolites—mainly lipids and acylcarnitines—increased during sleep deprivation. Furthermore, serotonin, tryptophan and taurine were enhanced, which might be one reason behind the anti-depressive effect reported for sleep deprivation [66]. This study specifically aimed to characterize circadian metabolomics variations in sleep-deprived humans. A circadian rhythmicity was observed in more than 50% of the cycling plasma metabolites, comprised of several metabolic classes, such as amino acids, biogenic amines, acylcarnitines, glycerophospholipids, and sphingolipids. Although a circadian rhythmicity was also observed in the absence of a night’s sleep, the amplitudes of all the metabolites were lower due to sleep deprivation [30].

In men, sleep deprivation caused a change of about half of the identified urinary metabolites. More specifically, it caused an increase of eight urinary metabolites (taurine, formate, citrate, 3-indoxyl sulfate, carnitine, 3-hydroxyisobutyrate, trimethylamine N-oxide and acetate), while another eight metabolites (dimethylamine, 4-deoxythreonic acid, creatinine, ascorbate, 2-hydroxyisobutyrate, allantoin, 4-deoxyeryhtronic acid and 4-hydroxyphenylacetate) decreased [65]. Furthermore, diurnal variations in the urinary metabolomics profiles were observed, emphasizing the need to consider the sampling time in the study of biomarkers.

Besides metabolomics studies conducted on plasma and urine samples, animal models have been used to obtain direct information about brain changes due to sleep deprivation. A recent study conducted in mice investigated the metabolome of the prefrontal cortex during sleep, sleep deprivation and spontaneous wakefulness [67]. Overall, 11 known metabolites were observed to be higher after wakefulness (in most of the cases, after spontaneous as well as enforced wakefulness), whereas no metabolite was found in higher concentrations after sleep. Metabolites that changed were mainly related to amino acid (glutamate, tryptophan) and energy metabolism (lactate, pyruvate), glucose metabolism (gluconate), pyrimidine pathway (orotate, uridine) and intermediates of the tricarboxylic cycle (succinate).

Metabolomics approaches have also been applied in a study investigating treatment responses. In a study conducted in rats submitted to sleep deprivation, the potential of extracts from Panax ginseng to restore serum and brain metabolic profiles was investigated [68]. Panax ginseng is a traditional Chinese medicine which is known for its multiple activities in the nervous system, such as improving cognition [70], but also to modulate effects of sleep deprivation on the blood system [71]. An extract from Panax ginseng has shown to improve cognition and behavior in a rodent model of sleep deprivation. Metabolomics analysis of brain tissues samples revealed a change in 39 metabolites due to sleep deprivation, whereas 40 metabolites changed in serum samples. Animals receiving ginsenoides showed a tendency to recover compared to the control group, which corresponded to recovering effects on behavioral and biochemical parameters. It has been suggested that ginsenoids are able to regulate dysfunctional energy, BCAA and adenosine metabolism as well as oxidative stress.

## 5. Sleep Restriction

Overall, sleep restriction better reflects the real life situation than total sleep deprivation. Several studies have investigated changes in the metabolic profile of individuals experiencing insufficient sleep (Table 3).

Reducing sleep has been shown to be associated with pronounced shifts in the lipid metabolome. Biomarkers of insufficient sleep were investigated in plasma samples of 16 normal-weight participants [72]. The individuals were subjected to three days with sufficient (9 h) sleep opportunity (Baseline), followed by a 5-day period of insufficient (5 h) sleep opportunity and final 5-day adequate (9 h) sleep opportunity. Metabolites differentiating insufficient vs. adequate sleep were mainly related to ATP-binding cassette transporters in lipid homeostasis, phospholipid metabolic process, plasma lipoprotein remodeling and sphingolipid metabolism. The changes in the lipid metabolome due to sleep restriction have also been observed by Weljie et al. [73]. In adult healthy individuals, four hours of sleep restriction for five days caused considerable alterations in lysophosphatidylcholines, triglycerides, phosphatidylcholines and acylcarnitines in the plasma of healthy individuals when compared to their baseline measurements. A single night of recovery could not restore the decreased level of long-chain acylcarnitines [73]. In addition, changes in amino acids were found, along with an increase in tryptophan and phenylalanine. A more acute sleep restriction paradigm demonstrated strong changes in lipid metabolism in healthy individuals with familial diabetic history [31]. A sleep opportunity of 5.5 h as compared to a baseline night of 8 h sleep opportunity caused changes in medium-chain fatty-acid (caproate), carnitine, lysolipid, cholesterol and bile acid metabolism, with an elevation of most plasma lipid metabolites. Furthermore, changes in carbohydrate and amino acid (i.e., tryptophan) metabolism were observed. Additionally, metabolites related to mitochondrial fatty-acid oxidation were found to be impacted by sleep restriction, showing an increase in acylcarnitines in healthy as well as diabetic individuals [74]. The detrimental effect of sleep restriction on metabolic health is also supported by metabolomics studies observing a decrease of high-density lipoproteins across two populations with subjective sleep insufficiency [75].

Rodent studies provide further insights into the effect of sleep restriction on energy metabolism and on the clinical complications of sleep restriction, including obesity, diabetes and neurological disorders. A study conducted in rats revealed altered hepatic profiles indicative of changes in hepatic energy metabolism, as well as alterations in nicotinamide adenine dinucleotide salvage pathways and transmethylation pathways. Findings suggest that sleep restriction reduced activity of the tricarboxylic acid cycle, which went along with a concomitant increase in lipogenesis [76].

## 6. Sleep Fragmentation

Sleep fragmentation refers to brief arousals occurring during sleep. Arousals occur frequently in patients with sleep disorders, such as OSA, but are also often induced by external factors, such as noise, bright light or high temperature during sleep [77]. Sleep fragmentation often goes along with excessive daytime sleepiness [78] and decreased daytime functioning [79].

Animal models are used to study the pathophysiological mechanism of sleep fragmentation as summarized in Table 4. The underlying mechanism of the detrimental effects of sleep interruption on cognitive functions remains largely unknown to date. A study conducted in mice submitted to chronical sleep interruption was undertaken to study its effect on serum metabolome and brain antioxidative metabolites as well as cognitive function [80]. Sleep fragmentation strongly affected cognitive function, which went along with an increase in the lipid peroxidation marker malondialdehyde in the brain, while the antioxidative enzymes superoxide dismutase and catalase decreased. Therefore, the cognitive decline might be related to excessive oxidative stress in the brain; however, perturbations of the systemic metabolism likely also contribute to the cognitive impairment. In this regard, 13 potential biomarkers related to amino acid, purine and lipid metabolism were identified in the serum. These metabolites included valine, choline, uric acid, allantoic acid, carnitines and retinoids. Furthermore, the chronic sleep interruption was associated with considerable weight loss.

Previous studies revealed that the hippocampus is especially vulnerable to sleep fragmentation, showing a reduction in hippocampal volume, learning and cognition deficits and reduced neurogenesis [81]. To reveal possible mechanisms by which sleep fragmentation impairs hippocampal neurogenesis, metabolomics might help to better understand the detrimental effects of sleep fragmentation on the brain. A recent study conducted in rodents pinpoints a metabolomic impact of sleep fragmentation on the brain. More specifically, Yoon et al. [77] analyzed metabolite profiles of the hippocampus in rats exposed to a 4- or 15-day sleep fragmentation protocol as compared to a 4- or 15-day exercise control group. Distinctive metabolic profiles were observed in the 15-day sleep fragmentation group, with most pronounced effects on the amino acid metabolism. More specifically, the alanine, aspartate, and glutamate metabolism pathways were identified as the common key pathways in rats subjected to sleep fragmentation for 15 days. An increase in tryptophan, myristoylcarnitine and palmitoylcarnitine was also observed, while methionine, glycerophosphocholine, adenosine monophosphate and hypoxanthine were decreased [77].

The role of the gut microbiome for mental and physical health disorders received increasing research interest in recent years [84,85]. A study conducted in rats investigated the effect of sleep fragmentation on their gut microbiome and fecal metabolome [82]. Rats subjected to a 28-day sleep fragmentation protocol showed a lower alpha-diversity and pronounced shifts in the fecal microbiome as well as changes in the fecal metabolome. Several fecal metabolites correlated with blood pressure, which might provide an insight into the reported link of sleep fragmentation and deleterious cardiovascular changes [86]. Furthermore, the effects of sleep fragmentation on the fecal metabolome and microbiome were investigated in mice [83]. A sub-chronic, five-day sleep disruption protocol caused a change in the level of bacterially-modified metabolites, such as bile acids, and caused a reduction of beneficial bacterial genera. Changes in the metabolic and microbial profile lasted at least four days after the end of the sleep disruption. Therefore, studies suggest an interplay between the microbiome and sleep-wake disorders which should be addressed in future studies.

## 7. Conclusions

This review on the application of metabolomics in the study of sleep disorders clearly suggests alterations in specific metabolic pathways which likely contribute to the association of sleep-wake disorders with other psychiatric disorders and medical conditions. Although differences in study populations and technologies used for the profiling of metabolites complicate comparison of these studies, a common pattern seems to arise in terms of changes in BCAA metabolic pathways. Furthermore, glucose and lipid metabolism, as well as antioxidative status, were found to be affected by changes in physiological sleep-wake cycles in several studies.

Overall, the observed results suggest that especially the BCAA metabolism is associated with the sleep-wake regulation. The three BCAAs L-leucine, L-valine, and L-isoleucine must be acquired through diet, as they cannot be synthesized de novo [87]. In several studies, BCAA concentrations were altered in sleep-wake disorders (Table 1, Table 2, Table 3 and Table 4). Elevated BCAA catabolism and glucose concentrations during the night have been suggested as initial signs of insulin resistance and have been implicated in the etiology of obesity, type 2 diabetes and metabolic syndrome [88,89].

In general, BCAAs are transported through the blood–brain barrier via the L-type or large amino acid transporter 1 (LAT1) to serve as a major nitrogen source for the brain [90]. As LAT1 is shared by several large amino acids, including BCAAs and aromatic amino acids (AAAs), these large amino acids compete for access to the LAT1 to be transported from the blood into the central nervous system [91]. The AAA tryptophan is a substrate for serotonin, while both tyrosine and phenylalanine are precursors for catecholamine (dopamine, norepinephrine and epinephrine) synthesis [92]. The three BCAAs are key elements in the de novo synthesis of glutamate [93]. An excess of BCAAs caused by sleep-wake disorders, can reduce the uptake of the serotonin precursor tryptophan and the catecholamine precursors tyrosine and phenylalanine; therefore, the decreased inhibitory neurotransmitters (serotonin and catecholamines) and increased excitatory neurotransmitters (glutamate and aspartate) might maintain the waking of the brain [68]. Elevation of BCAAs during nighttime has also been linked to daytime fatigue, likely due to their capacity to reduce the transport of tryptophan through the blood–brain barrier, finally affecting serotonin levels [94]. This is supported by an observed reduction of daytime tryptophan and phenylalanine in patients with insomnia [28]. As serotonin is significantly involved in the sleep/wake regulation [95], a decreased serotonin concentration due to reduced uptake of its precursor tryptophan has been speculated to explain the impaired ability to initiate and maintain sleep [28].

While on the one hand, elevation of BCAAs in healthy individuals can negatively affect the sleep/wake rhythmicity and metabolic health, on the other hand, supplementation of BCAAs has been suggested as a viable therapy for treating sleep/wake disturbances in individuals suffering from traumatic brain injury [19]. Traumatic brain injury can result in disturbed sleep, whereby supplementation of BCAAs in mice subjected to a mild brain injury has been shown to improve quality of sleep [19]. As BCAAs are essential for de novo cerebral synthesis of glutamate and γ-aminobutyric acid (GABA), BCAA supplementation is expected to be able to restore pools of releasable vesicular glutamate, which have been reported to decrease after traumatic brain injury [96]. A decreased concentration of glutamate leads to reduced excitatory inputs onto the wake-promoting system [97]. Studies conducted in rodents suggest that BCAA supplementations can restore normal cortical excitability through direct action on orexin neurons, which are a critical components of the sleep/wake regulatory system [98]. Moreover, BCAA supplementation has been shown to restore cognitive function after impairment due to traumatic brain injury in rodents [97].

Despite the overwhelming consensus that metabolomics bears potential for biomarkers in the management of sleep-wake disorders, there are still limitations in the existing metabolomics work. A major limitation is that existing studies mainly focused on relatively small sample sizes under limited clinical conditions [64]. Although several studies were already conducted with human subjects, many of them were limited to healthy individuals submitted to specific sleeping regimens. Thus, further studies with larger sample sizes of patients with sleeping disorders are needed to gain deeper knowledge on the metabolomic changes that exist in clinical practice. A further limitation is that although metabolomics shows potential in the diagnosis of sleep disorders, it cannot replace clinical assessments, such as polysomnography measurements, to date. One limitation that precludes the application of metabolomics for diagnosis is that there is a lack of normative values of metabolite concentrations [99]. A further aspect is that the metabolic profile can be considerably affected by various variables, such as gender, nutrition, medical comorbidities or medication, as well as diurnal variations, which require further research [100]. Furthermore, studies conducted on the most relevant sampling substrates for identifying biomarkers of mental disorders, brain tissues, have solely been conducted using animal models as brain-derived samples from humans are commonly only available at autopsy [101]. Although these animal models are helpful to study causal links between mental disorders and the affected molecular pathways, they face several limitations, as reviewed recently [102]. A further important limitation is that studies investigating prognostic utility are missing so far. In general, there has been only very limited research on specific treatments’ effects on the metabolome until now. Therefore, research is needed to elucidate whether metabolomics holds potential to predict treatment responses. Overall, further studies are needed, examining, for example, the severity and subtypes of sleep-wake disorders and treatment of patients with sleeping disorders in the context of metabolite levels.

Finally, it can be concluded that metabolomics provides important insights into the metabolic pathways linked to sleep disorders; however, the usability in clinical practice is limited to date as no references for normal ranges of metabolites exist.

## Figures and Tables

**Table 1:** Identified insomnia biomarkers.

Subject	Sample	Analytical Platform	Metabolites	Pathways/Functions	Reference
Humans	Serum	NMR ^1^	Isoleucine, valine, lysine, alanine, serine, proline, phenylalanine, tyrosine, acetate, 3-hydroxybutyrate, citrate, dimethylamine, dimethylglycine, ornithine, creatinine, 2-hydroxyvalerate, 2-oxoisocaproate, 3-methyl-2-oxovalerate, propylene glycol, N-acetyl metabolites, methylhistidine, lactate, succinate	Amino acid metabolism, energy (glucose) metabolism	[28]
Humans	Plasma	LC-MS ^2^	Isoleucine, leucine, proline, arginine, ornithine, octadecanoylcarnitine, glycerophospholipids, lysophosphoatidylcholines, sphingolipids	Amino acid metabolism, lipid metabolism	[33]
Humans	Plasma	LC-MS/MS ^3^, GC-MS ^4^	Isoleucine, leucine, valine, gamma glutamyl, bile acids, carnitines, fatty acids	Amino acid metabolism, lipid metabolism, energy metabolism	[29]

^1^ NMR, nuclear magnetic resonance spectroscopy. ^2^ LC-MS, liquid chromatography–mass spectrometry. ^3^ LC-MS/MS, liquid chromatography-tandem mass spectrometry. ^4^ GC-MS, gas chromatography–mass spectrometry.

**Table 2:** Possible biomarkers identified in sleep-deprived individuals.

Subject	Sample	Analytical Platform	Metabolites	Pathways/Functions	Reference
Humans	Plasma	LC-MS ^1^	Serotonin, tryptophan, taurine, acylcarnitines, glycerophospholipids, sphingolipids	Amino acid metabolism, lipid metabolism, neurotransmitter metabolism	[30]
Humans	Urine	NMR ^2^	Taurine, formate, citrate, 3-indoxyl sulfate, carnitine, 3-hydroxyisobutyrate, trimethylamine-N-oxide, acetate, dimethylamine, 4-deoxythreonic acid, creatinine, ascorbate, 2-hydroxyisobutyrate, allantoin, 4-deoxyeryhtronic acid, 4-hydroxyphenylacetate	Neurotransmitter metabolism, fatty acid metabolism, energy metabolism, amino acid metabolism	[65]
Mice	Brain	UPLC-HRMS ^3^	Glutamate, tryptophan, lactate, pyruvate, glucose metabolism, orotate, uridine, succinate	Amino acid metabolism, energy metabolism, glucose metabolism, pyrimidine pathway, tricarboxylic acid cycle	[67]
Rats	Brain	GC-MS ^4^	Valine, leucine, isoleucine, tyrosine, cysteine, threonine, serine, methionine, 4-hydroxyproline, glycerol 3-phosphate, 3-hydroxybutyric acid, glutamic acid, aspartic acid, adenosine, cytidine monophosphate, uracil, inosine, hypoxanthine, xanthine, lactic acid, fructose, palmitoleic acid	Energy metabolism, adenosine metabolism, amino acid metabolism, neurotransmitter metabolism, oxidative stress	[68]
Rats	Serum	GC-MS	Valine, leucine, alanine, cysteine, glycine, threonine, methionine, serine, 4-hydroxyproline, glycerol 3-phosphate, 3-hydroxybutyric acid, glutamic acid, aspartic acid, stearic acid, fructose, glutamic acid, ethanolamine, serotonin, cholesterol, inositol phosphate	Energy metabolism, adenosine metabolism, amino acid metabolism, neurotransmitter metabolism, oxidative stress	[68]
Rats	Serum	NMR	Lipoproteins, triglycerides, isoleucine, valine, choline, phosphorylcholine, total fatty acids, saturated fatty acids, unsaturated fatty acids, monounsaturated fatty acids, polyunsaturated fatty acids, components, glucose, insulin	Fatty acid metabolism, glucose metabolism	[69]

^1^ LC-MS, liquid chromatography–mass spectrometry. ^2^ NMR, nuclear magnetic resonance spectroscopy. ^3^ UPLC-HRMS, ultra-performance liquid chromatography–high resolution mass spectrometry. ^4^ GC-MS, gas chromatography–mass spectrometry.

**Table 3:** Possible biomarkers identified in sleep restricted individuals.

Subject	Sample	Analytical Platform	Metabolites	Pathways/Functions	Reference
Humans	Plasma	LC-MS/MS ^1^	Sphingolipids (ceramide 40:2, ceramide d41:2, sphingomyelin 43:2, sphingomyelin d33:2), lysophosphatidylcholine 18:3, phosphatidylcholine 40:5	ATP-binding cassette transporters in lipid homeostasis, phospholipid metabolic process, plasma lipoprotein remodeling, sphingolipid metabolism	[72]
Humans	Plasma	GC-MS ^2^	Lysophosphatidylcholines (14:0, 16:1, 17:0), phosphatidylcholines (32:1, 36:6, 38:4, 38:2, 38:3), acylcarnitines (C5:0, C10:0, C12:0), ceramides, diacylglycerol 36:3, oxalic acid	Lipid metabolism, fatty-acid metabolism, amino acid metabolism	[73]
Rats	Plasma	GC-MS	Lysophosphatidylcholines, phosphatidylcholines, diacylglycerol 36:3, leucine, valine, oxalic acid, sucrose	Lipid metabolism, fatty-acid metabolism, amino acid metabolism	[73]
Humans	Plasma	LC-MS/MS	N-acetylthreonine, histidine, glutaroyl carnitine, phenyllactate, C-glycosyltryptophan, serotonin, isoleucine, mannose, 1,6-anhydroglucose, glycocholenate sulfate, cholesterol, beta-sitosterol, 7-alpha-hydroxy-3-oxo-4-cholestenoate, pantothenate, gamma-CEHC, benzoate, piperine	Lipid metabolism, amino acid metabolism, carbohydrate metabolism	[31]
Humans	Plasma	LC-MS/MS	Tetradecenoyl-L-carnitine (C14:1), octadecanoyl-L-carnitine (C18:1), octadecadienyl-L-carnitine (C18:2)	Energy metabolism (mitochondrial fatty-acid oxidation)	[74]
Rats	Liver	LC-MS ^3^	Nicotinamide adenine dinucleotide, Nicotinamide adenine dinucleotide phosphate, N-methylnicotinamide, nicotinamide riboside, histidine, glutamine, adenine, adenosine, AMP, guanosine, glutamine, methionine, S-adenosyl homocysteine, S-adenosyl methionine, methionine sulfoxide N1-methyl-2-pyridone-5-carboxamide, N1-methyl-3-pyridone-4-carboxamide, serine, aspartate, adenosine triphosphate, urea, xanthine, xanthosine, aconitate, citrate, isocitrate, serine	Energy metabolism (tricarboxylic acid cycle), nicotinate and nicotinamide metabolism, ammonia recycling, urea cycle, methionine metabolism	[76]

^1^ LC-MS/MS, liquid chromatography-tandem mass spectrometry. ^2^ GC-MS, gas chromatography–mass spectrometry. ^3^ LC-MS, liquid chromatography–mass spectrometry.

**Table 4:** Possible biomarkers in sleep fragmentation.

Subject	Sample	Analytical Platform	Metabolites	Pathways/Functions	Reference
Rats	Brain	LC-MS ^1^	Alanine, aspartate, and glutamate, methionine, tryptophan, myristoylcarnitine, palmitoylcarnitine, glycerophosphocholine, adenosine monophosphate, hypoxanthine	Amino acid metabolism	[77]
Mice	Serum	LC-MS	Valine, choline, uric acid, allantoic acid, carnitines, retinoids	Amino acid metabolism, purine metabolism, lipid metabolism	[80]
Mice	Brain	LC-MS	Malondialdehyde, superoxide dismutase, decreased	Oxidative stress	[80]
Rats	Feces	NMR ^2^	UDP-glucose, 3-hydroxyisovalerate, glutamine, inosine	Glucose metabolism	[82]
Mice	Feces	LC-MS/MS ^3^	Bile acids, urobilin, cholic acid, alanine, glutamine, lysine, valine, cysteine, lysine, asparagine, isoleucine	Lipid metabolism, glucose metabolism, bile acid metabolism	[83]

^1^ LC-MS, liquid chromatography–mass spectrometry. ^2^ NMR, nuclear magnetic resonance spectroscopy. ^3^ LC-MS/MS liquid chromatography–tandem mass spectrometry.

## Abbreviations

AAA	Aromatic amino acid
BCAA	Branched-chain amino acid
CPAP	Continuous positive airwave pressure
DSM	Diagnostic and statistical manual of mental disorders
GABA	γ-aminobutyric acid
GC-MS	Gas chromatography–mass spectrometry
LAT1	Large amino acid transporter 1
LC-MS	Liquid chromatography–mass spectrometry
LC-MS/MS	Liquid chromatography–tandem mass spectrometry
NMR	Nuclear magnetic resonance spectroscopy
NREM	Non–rapid eye movement
OSA	Obstructive sleep apnea
PRISMA	Preferred reporting items for systematic reviews and meta-analyses
REM	Rapid eye movement
SWS	Slow wave sleep
UPLC-HRMS	Ultra-performance liquid chromatography–high resolution mass spectrometry
